# Efficient biochemical production of acetoin from carbon dioxide using *Cupriavidus necator* H16

**DOI:** 10.1186/s13068-019-1512-x

**Published:** 2019-06-28

**Authors:** Carina Windhorst, Johannes Gescher

**Affiliations:** 1 Department of Applied Biology, Institute for Applied Biosciences, Karlsruhe Institute of Technology, Karlsruhe, Germany; 2 Institute for Biological Interfaces, Karlsruhe Institute of Technology, Eggenstein-Leopoldshafen, Germany

**Keywords:** *Cupriavidus necator* H16, *Ralstonia eutropha* H16, Acetoin, Autotroph, Platform chemical, Polyhydroxybutyrate

## Abstract

**Background:**

*Cupriavidus necator* is the best-studied knallgas (also termed hydrogen oxidizing) bacterium and provides a model organism for studying the production of the storage polymer polyhydroxybutyrate (PHB). Genetically engineered strains could be applied for the autotrophic production of valuable chemicals. Nevertheless, the efficiency of the catalyzed processes is generally believed to be lower than with acetogenic bacteria. Experimental data on the potential efficiency of autotrophic production with *C. necator* are sparse. Hence, this study aimed at developing a strain for the production of the bulk chemical acetoin from carbon dioxide and to analyze the carbon and electron yield in detail.

**Results:**

We developed a constitutive promoter system based on the natural PHB promoter of this organism. Codon-optimized versions of the acetolactate dehydrogenase (*alsS*) and acetolactate decarboxylase (*alsD*) from *Bacillus subtilis* were cloned under control of the PHB promoter in order to produce acetoin from pyruvate. The production process’s efficiency could be significantly increased by deleting the PHB synthase *phaC2*. Further deletion of the other PHB synthase encoded in the genome (*phaC1*) led to a strain that produced acetoin with > 100% carbon efficiency. This increase in efficiency is most probably due to a minor amount of cell lysis. Using a variation in hydrogen and oxygen gas mixtures, we observed that the optimal oxygen concentration for the process was between 15 and 20%.

**Conclusion:**

To the best of our knowledge, this study describes for the first time a highly efficient process for the chemolithoautotrophic production of the platform chemical acetoin.

**Electronic supplementary material:**

The online version of this article (10.1186/s13068-019-1512-x) contains supplementary material, which is available to authorized users.

## Background

Biotechnology can be the key to the advent of a bioeconomy as this technology can use renewable resources for sustainable production processes. In this study, we focused on the production of acetoin using *Cupriavidus necator* H16 as biocatalyst. *C. necator* is a model organism for both polyhydroxybutyrate (PHB) production and chemolithoautotrophic growth. Its status as a model organism is also due to the availability of its complete genome sequence [1, 2] and its genetic tractability. The organism grows heterotrophically with fructose, *N*-acetylglucosamine, gluconate, fatty acids, and other compounds as electron donors and carbon sources using oxygen, nitrate, nitrite, or dimethyl sulfoxide (DMSO) as electron acceptors. Under chemolithoautotrophic growth conditions, *C. necator* can use carbon dioxide (CO_2_) as a carbon source, hydrogen (H_2_) as an electron donor, and oxygen (O_2_) as an electron acceptor. Due to its metabolic versatility and accessible high cell densities, *C. necator* has often been suggested to be a good candidate for biotechnological processes. Consequently, multiple studies present strains that produce a large variety of chemicals ranging, for example, from α-humulene (over isopropanol, methyl ketones, and isobutanol) to 3-methyl-1-butanol [3–7]. However, studies evaluating the efficiency of autotrophic production processes for platform chemicals are rather sparse. Przybylski et al. [8], for instance, presented a strain for the autotrophic production of the methacrylate precursor 2-hydroxyisobutyric acid. The strain produced the C4 compound with an efficiency of 0.03 mol per mol of CO_2_ consumed. Krieg and colleagues [3] aimed for the production of the C15 compound, α-humulene. The complexity of this substance was most probably the reason why the carbon efficiency was 30-fold lower compared to hydroxyisobutyrate production [3].

Although the carbon efficiency might not be high enough at this point, it was suggested that knallgas bacteria, such as *C. necator*, could be the key for the production of more complex end products from syngas streams [9]. Currently, acetogens are mostly used for syngas fermentation processes. The organisms are very efficient catalysts, especially due to Wood–Ljungdahl-based CO_2_ fixation and use of CO_2_ as a carbon source. Still, acetogens growth is rather energy limited compared to *C. necator.* The ΔG° value of an acetogen growing with hydrogen and CO_2_ and producing acetate is − 111 kJ mol^−1^. A knallgas bacterium using hydrogen as electron donor and oxygen as electron acceptor shows a ΔG° value of − 474 kJ mol^−1^. Hence, the possibilities to redirect this bacterium’s central metabolism toward valuable end products will be easier. Moreover, acetogens are often difficult to genetically manipulate, which hampers the development of strains with a metabolism tailored toward the production of the desired end products. A recent study suggested that heterologous expression of a CO reductase could render *C. necator* capable of using CO as electron carrier to a certain extent [10]. The latter would allow to use a number of gas streams as substrates that originate, for instance, from steel production and contain CO in varying concentrations—a production niche that was exclusively exploited using acetogens to date. A benefit and challenge for the autotrophic production using *C. necator* are that the organism uses the Calvin cycle for CO_2_ fixation. The benefit is that the cycle funnels carbon as a C3 compound into glycolysis and/or gluconeogenesis, which allows the production of complex compound branching, for instance, from the central intermediate pyruvate. The disadvantage is that the unwanted oxygenase activity of the key enzyme ribulose-1,5-bisphosphate carboxylase/oxygenase (RuBisCO) can divert a substantial amount of electrons toward the reduction in oxygen instead of CO_2_.

The *C. necator* wild-type strain is naturally producing the storage polymer PHB throughout all growth conditions and nutrition modes [11, 12]. The PHB concentration can be increased via limitation of nitrogen, phosphorus, magnesium, or sulfur and a surplus of the carbon source in the medium. Three enzymes are involved in PHB production. First, two molecules of acetyl-coenzyme A are converted to one molecule 3-acetoacetyl-CoA by the β-ketothiolase PhaA. The nicotinamide adenine dinucleotide phosphate (NADPH)-dependent acetoacetyl-CoA reductase, PhaB, catalyzes the formation of 3-hydroxy-butyryl-CoA while the PHB synthase PhaC polymerizes this compound into PHB. The genome sequence of *C. necator* consists of multiple putative copies of genes encoding PhaA (38 genes), PhaB (15 genes), and PhaC (two genes) [2].

This study focused on the production of the platform chemical acetoin. Acetoin was proposed to be one of the top 30 value-added chemicals that can be produced from biomass [13]. Acetoin has a wide spectrum of potential applications due to its character as a platform chemical [14] and is also a common additive in food industry due to its buttery flavor. On average, each person consumes 2 to 4 mg acetoin per day [15], which results in an annual global consumption of several thousand tons of acetoin. Acetoin is currently produced using fossil feedstocks and chemical processes [14]. Bacteria produce acetoin via two steps: (1) the first step is an acetolactate synthase (*alsS*)-catalyzed condensation of two pyruvate molecules to acetolactate and the concomitant release of one CO_2_ molecule, and (2) in the second step, acetolactate decarboxylase (*alsD*) activity leads to acetoin formation by releasing a second CO_2_ molecule. Different bacterial strains naturally produce acetoin; the most prominent producer is *Bacillus subtilis*. An ultraviolet (UV)-mutagenized *B. subtilis* strain produced more than 50 g/l acetoin from 150 g/l glucose, which correlates to an acetoin yield of 73.5%, considering that a maximum of 1 mol of acetoin can be produced from 1 mol of glucose [16]. The application of genetically manipulated bacterial strains can increase the production yield to up to 96% (mol/mol) of the theoretical maximum under oxic conditions [17]. Under anoxic conditions, Förster et al. [18] and Bursac et al. [19] showed production yields in minimal medium with *Escherichia coli* and *Shewanella oneidensis* of up to 90% and 86% (mol/mol), respectively. To the best of our knowledge, no study has thus far elucidated the possibility of producing acetoin under chemolithoautotrophic conditions.

Consequently, this study aimed to develop a bacterial strain for the efficient production of acetoin from CO_2_. In order to reach this goal, we determined the optimal oxygen concentration for lithoautotrophic growth and studied the influence of different promoter systems and deletions of the PHB synthases on the acetoin production process. In the end, we engineered a strain with superior CO_2_ and electron efficiencies compared to the *C. necator* wild-type strain.

## Materials and methods

### Bacterial strains and growth conditions

All bacterial strains and plasmids used in this study are listed in Additional file 1: Table S1. *C. necator* strains were cultivated in lysogeny broth (LB) or in minimal media (MM) 81 (DSMZ) (Additional file 1: Table S2) at 30 °C. Minimal medium was supplemented for heterotrophic growth with 10 mM fructose or acetoin or for autotrophic growth with a gas mixture of 80% H_2_:5% CO_2_:15% O_2_. *E. coli* strains were cultivated in LB at 37 °C. If necessary, kanamycin (50 µg/ml in water) or tetracycline (15 µg/ml in DMSO) was added. For promoter induction, l-arabinose (1 µM up to 3 mM) or l-rhamnose (1 µM–1.5 mM) was added to the medium. *E. coli* WM3064 was grown in medium containing 0.3 mM diaminopimelic acid (DAP). If necessary, the medium was supplemented with 2% agar.

All experiments with explosive gas mixtures were conducted in fume hoods in order to assure a constant discharge of the explosive gas mixtures and with explosion protection devices.

### Strain and plasmid construction

DNA was amplified with Hifi Polymerase (Nippon Genetics Europe, Dueren/Germany) in preparative polymerase chain reactions (PCRs) or with MangoMix (Bioline, Luckenwalde/Germany) in diagnostic PCRs. Gel purifications of DNA products were carried out using the Wizard^®^ SV Gel and PCR Clean-Up System (Promega, Mannheim/Germany). The Wizard^®^Plus SV Minipreps DNA Purification System (Promega, Mannheim/Germany) was used for plasmid isolation. Restriction enzymes were purchased from New England Biolabs (Frankfurt/Germany). Primers were ordered from Sigma-Aldrich/Merck (Darmstadt/Germany). Sequencing of constructs was conducted by Eurofins Genomics (Ebersberg/Germany) and aligned with the expected sequences using the CLC main workbench (Version 8.0, Qiagen, Aarhus/Denmark).

The plasmids for gene deletions were constructed via amplification of approximately 500 bp upstream and downstream of the target gene (primers: Δ*acoABC*-up: 2596 and 2581, down: 2636 and 2597; Δ*phaC1*-up: 2682 and 2685, down: 2684 and 2683; Δ*phaC2*-up: 2687 and 2689, down: 2688 and 2690, see Additional file 1: Table S3). The PCR products had overlaps to the linearized pMQ150 plasmid (*Eco*RI and *Bam*HI) and were assembled using the Gibson Assembly [20]. Afterward, the dialyzed Gibson Assembly mix was transformed in *E. coli* WM3064. The strain carrying the correct plasmid was used as the donor strain for conjugation. The donor and recipient strains were streaked out on LB agar plates with diaminopimelic acid (DAP). After 24 h, the cell mixture was transferred to LB agar plates with antibiotic and without DAP. After an incubation time of 2 to 3 days, the colonies were tested for plasmid integration using diagnostic PCR. For the second cross, cells were first cultivated for 24 h in liquid LB without NaCl containing 25% sucrose and were then streaked onto agar plates containing the same medium. Verification was performed via diagnostic PCR and subsequent sequencing.

The *alsSD* genes from *B. subtilis* PY79 were selected for the expression experiment. The genes were codon optimized (see Additional file 2: Sequence *alsS* and Additional file 3: Sequence *alsD*) according to the codon usage of *C. necator* H16 [21] and ordered as two strings (*alsSD* I and *alsSD* II). PCR products from the strings (primers: string *alsSD* I: 2588 and 2657; string *alsSD* II: 2589 and 2658) were assembled in a two-step PCR using primers 2657 and 2658 and cloned via Gibson Assembly in plasmid pKRrha-eGFP (cut by *Nde*I-HF and *Bsr*GI-HF) resulting in plasmid pKRrha-*alsSD*.

For the construction of promoter exchange plasmids, the plasmid pKRrha-*alsSD* was amplified by two inverse PCRs without amplifying *rhaR* and *rhaS* (fragment I: primers 2701 and 188; fragment II: primers 2694 and 2693). The arabinose-inducible promoter and *araC* were amplified from the plasmid pBAD (using primers 2697 and 2698). A 2000 bp fragment upstream of the *phaCAB* operon in the genome of *C. necator* H16 wild type was amplified to gain the hypothetical promoter of the PHB-synthesis gene cluster *phaCAB* (primers 2695 and 2696). The vector fragments I and II were assembled with the ara promoter, and the hypothetical PHB promoter resulted in pKRara-*alsSD* and pKRphb-*alsSD*, respectively. The plasmids were conjugated into the recipient strains as previously described.

### Quantification of substrate and products

Samples were taken at different time points and analyzed with regard to the optical density (OD_600_) as well as the fructose and acetoin concentration. The optical density was measured in a Genesys 20 spectrophotometer (Thermo Spectronic/Thermo Fisher Scientific, Darmstadt/Germany) or an Ultrospec 10 Cell Density Meter (Biochrom/Merck, Darmstadt/Germany).

In order to determine the fructose concentration, 150 µl samples were filtered through a 0.2 µm polytetrafluoroethylene (PTFE) membrane (VWR, Darmstadt/Germany) and mixed in a 96 well microtiter plate with 15 µl 0.5 M sulfuric acid and measured via high-performance liquid chromatography ([HPLC] Dionex UltiMate 3000 HPLC; Thermo Scientific, Waltham, MA) according to Bursac et al. [19].

Concentrations of H_2_, O_2_, N_2_, and CO_2_ gases were measured via gas chromatography (490 Micro GC, Agilent Technologies Deutschland, Waldbronn/Germany) and analyzed using the software Agilent OpenLAB CDS (EZChrom Edition) (Agilent Technologies Deutschland, Waldbronn/Germany). The method was: (1) 5 s stabilizing time; (2) 20 s sample time; (3) 110 °C sample line and injector temperatures; (4) 50 ms injector time; (5) first column: 10 m MS5A, argon as carrier gas, 70 °C column temperature, 150 kPa column pressure; and (6) second column: 10 m PPQ, helium as carrier gas, 45 °C column temperature, 150 kPa column pressure.

The acetoin concentration was measured spectrophotometrically using the Voges–Proskauer reaction as previously described [22, 23].

### Optimal inductor concentration

In order to determine the optimal inductor concentration for the inducible promoters, cells were precultured in LB. Test tubes were filled with 5 ml MM 81 containing 10 mM fructose. The starting OD_600_ was adjusted to 0.6, and different inductor concentrations (arabinose: 0, 1, 5, 10, 50 µM, and 0.1, 0.5, 1, 1.5, 3, 5, 10 mM; rhamnose: 0, 1, 5, 10, 50 µM and 0.1, 0.5, 1, 1.5 mM) were added to the medium. OD_600_ values were measured, and samples were taken for the quantification of acetoin concentrations (Additional file 4: Figures S1 and S2).

### PHB analysis

Two methods were applied to analyze PHB production: (1) photometric measurement via Nile Red staining and (2) fluorescence microscopy. For the Nile Red staining, the different deletion mutants were cultivated for 20 h. One-hundred fifty microliters of an OD_600_ 1.0 cell culture were mixed with 50 µl Nile Red (2 µg/ml in DMSO) and incubated in darkness for 30 min at room temperature. The samples’ cell densities were measured at 600 nm, while Nile Red was quantified using excitation and emission wavelengths of 530 and 575 nm (infinite M200 Pro, Tecan, Maennedorf/Switzerland).

For microscopic imaging, 1 ml culture was harvested and resuspended in 150 µl phosphate-buffered saline (PBS) consisting of 138 mM NaCl, 2.7 mM KCl, 10 mM Na_2_HPO_4_, and 1.7 mM KH_2_PO_4_. After an incubation with 50 µl Nile Red (2 µg/ml in DMSO) for 30 min at room temperature in the dark, 40 µl 4′,6-diamidino-2-phenylindole (DAPI) at a concentration of 10 µg/ml in water was added, and the samples were incubated for another 10 min. The cells were washed with 150 µl PBS. Samples were viewed under a Leica DM 5500B microscope (objective lens 100×: HCX PL FLUOTAR, 1.4, oil immersion; eyepiece 10×: HC PLAN s [25] M), and images were taken with a Leica DFC 360 FX camera and analyzed with the corresponding Leica LAS AF Lite software.

### Heterotrophic acetoin production

Cells were precultured in LB. Acetoin production was achieved in 100 ml MM 81 medium containing 10 mM fructose. The starting OD_600_ was 0.6. Samples were taken to measure OD_600_, acetoin, and fructose concentrations and to perform RNA isolation in addition to subsequent real-time qPCR.

### qPCR

qPCR reactions were conducted in order to determine *alsS* and *alsD* mRNA levels. The housekeeping gene, *gyrB*, served as positive control, while a non-transcribed area was used as the negative control (Additional file 1: Table S4). RNA was isolated from pooled triplicates corresponding to a cell number of an OD_600_ of 1.0 (Qiagen RNeasy Mini Kit, Hilden/Germany). DNA was hydrolyzed using the Ambion DNA-free DNase Treatment and Removal Reagents kit (Thermo Fisher Scientific, Darmstadt/Germany). Five microliters of DNA-free RNA samples (20–25 ng/µl) were transcribed into cDNA (iScript Select cDNA, Bio‐Rad Laboratories, Munich, Germany). 2 µl cDNA were used as template (SsoAdvanced™ Universal SYBR^®^ Green Supermix, Bio‐Rad Laboratories, Munich, Germany). qPCR reactions were conducted and analyzed in triplicates using the CFX96™ Real‐Time PCR Detection System (Bio‐Rad Laboratories, Munich, Germany).

### Determination of the optimal oxygen concentration

The optimal oxygen concentration for growth was determined using four Biostat A fermenters (Sartorius, Goettingen/Germany) with a continuous gas supply of H_2_, CO_2_, and O_2_ (Additional file 4: Figure S3). The composition of the gas mixture was adjusted using the gas mixer EL-FLOW (Bronkhorst, Ruurlo/Netherlands) and the software FlowDDE and FlowView (Bronkhorst, Ruurlo/Netherlands). Different gas mixtures and different gas flows were used (Additional file 1: Table S5). The fermenters were inoculated to a starting OD_600_ of 0.1. The stirring speed was adjusted to 400 rpm and the temperature to 30 °C. The OD_600_ was measured for 10 h after which the gas concentration was altered to increase or decrease the oxygen concentration. The cells then grew in the new gas mixture for 14 h. The experiments were performed with *C. necator* H16 wild-type cells in 1 l of MM 81 medium without any carbon source.

### Autotrophic acetoin production

Cells were precultured in LB. One-liter anoxic Schott bottles with a gas-tight lid were flushed with the autotrophic gas mixture with a flow of 6 l/h for 2.5 h. Thereafter, the gas mixture in the bottle was measured with the Micro GC. Fifty microliters of MM 81 medium with a starting OD_600_ of 4.5 were injected. The gas pressure was balanced with air to a pressure of 1 bar. Samples were taken for OD_600_, and acetoin detection using a syringe and the gas mixture was measured by Micro GC analysis. The experiments were stopped at a CO_2_ concentration < 0.05%.

For acetoin production with a continuous gas flow, fermenters were inoculated to an OD_600_ of 2.0. Every other day, antibiotic was added to ensure that the plasmid was retained by the cells. The pH was adjusted to 7.0 with 1 M NaOH. Samples were taken to determine the OD_600_ and acetoin concentration.

## Results

### Strain development toward heterotrophic acetoin production

**Fig. 1:**
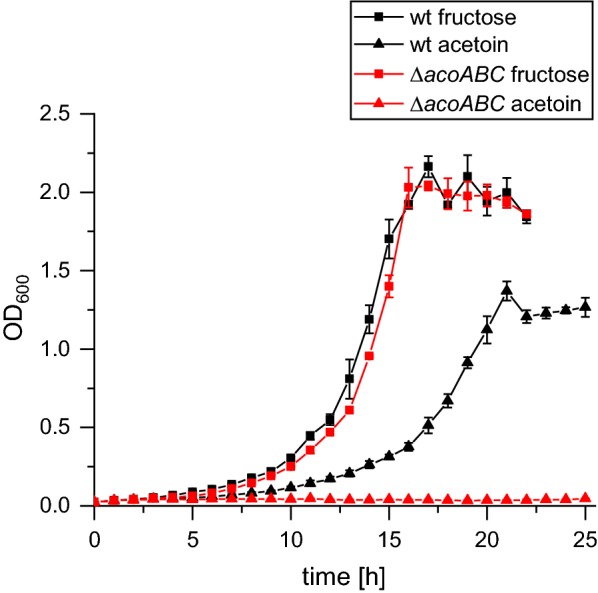
Growth of *C. necator* H16 wild type and *C. necator* H16_Δ*acoABC* with 10 mM fructose or acetoin as the sole carbon source

The aim of this study was to develop efficient heterotrophic and autotrophic production of the platform chemical acetoin. An outline of the central carbon metabolism of *C. necator* H16 is given in Additional file 4: Figure S4. As depicted in this figure, the strain is able to metabolize acetoin to acetaldehyde and acetyl-CoA [24]. Hence, the production of acetoin would potentially be hampered by its concurrent consumption. Therefore, the corresponding operon for acetoin consumption encoding the genes *acoABC* was deleted from the genome of the organism. In order to verify a successful deletion, both strains, *C. necator* H16 wild type and *C. necator* H16_Δ*acoABC*, were cultivated with 10 mM fructose or 10 mM acetoin as the sole carbon source (Fig. 1). Growth of both strains with 10 mM fructose did not show a significant difference. The wild-type strain grew with 10 mM acetoin, but the growth was slower than with fructose and reached a lower final optical density (OD_600_) of 1.2. As expected, the deletion strain could not thrive on acetoin as a carbon and electron source.

**Fig. 2:**
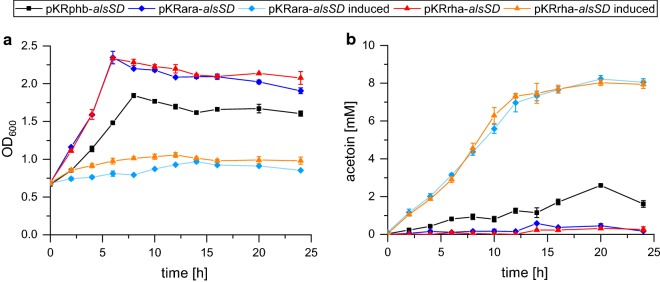
Comparative acetoin production of the three different strains in the inductor’s presence and absence. The figures show growth (**a**) and acetoin production (**b**). *C. necator* H16_Δ*acoABC*_pKRphb-*alsSD* is depicted by a black line. The control experiments with an uninduced *C. necator* H16_Δ*acoABC*_pKRara-*alsSD* strain are shown in dark blue, while experiments with 1.5 mM arabinose are indicated in light blue. Experiments with uninduced *C. necator* H16_Δ*acoABC*_pKRrha-*alsSD* are indicated in red. Experiments under induction with 1 mM rhamnose are indicated in orange

In order to achieve successful acetoin production, a plasmid with codon-optimized versions of the *alsSD* genes from *B. subtilis* PY79 was constructed (Additional file 4: Figure S4). The genes were placed under control of three different promoters: (1) plasmid, pKRrha-*alsSD*, had a rhamnose-inducible promoter; (2) pKRara-*alsSD*, an arabinose-inducible promoter; and (3) the last plasmid, pKRphb-*alsSD*, had the constitutively expressed promoter of the *phaCAB* operon, which is responsible for polyhydroxybutyrate (PHB) production [12, 25]. The optimal inductor concentrations for the inducible promoter systems (arabinose and rhamnose) were experimentally identified as 1.5 mM arabinose (Additional file 4: Figure S1) and 1 mM rhamnose (Additional file 4: Figure S2), respectively. All acetoin production experiments were inoculated to a starting OD_600_ of 0.6. The strains harboring the rhamnose- and arabinose-inducible plasmids grew to a maximal OD_600_ of 1.0 in medium containing the respective inductor (Fig. 2). The uninduced strains *C. necator* H16_Δ*acoABC*_pKRrha-*alsSD* and *C. necator* H16_Δ*acoABC*_pKRara-*alsSD* reached a maximum OD_600_ of 2.3 after 6 h. The strain with the constitutively expressed *alsSD* genes, *C. necator* H16_Δ*acoABC*_pKRphb-*alsSD*, had a final OD_600_ of 1.8 after 8 h. Acetoin production showed an inverse behavior with respect to growth. Acetoin production of 0.3 and 0.4 mM was measured for the uninduced Δ*acoABC* strains carrying the pKRrha-*alsSD* and pKRara-*alsSD* plasmids, respectively. The same strains cultivated with inducer reached a final acetoin concentration of up to 8.1 mM. The constitutive expression of the *alsSD* genes in *C. necator* H16_Δ*acoABC*_pKRphb-*alsSD* led to an acetoin production of 2.55 mM. In comparison with the consumed fructose, an acetoin yield of 89.9% and 87.1% of the theoretical maximum (mol/mol) was gained with the induced strains harboring plasmids pKRrha-*alsSD* and pKRara-*alsSD*, respectively. The acetoin yield of the strain containing the *alsSD* genes under *phaC1* promoter control was with 26.2% (mol/mol) significantly lower.

### Influence of PHB synthases on acetoin production

**Fig. 3:**
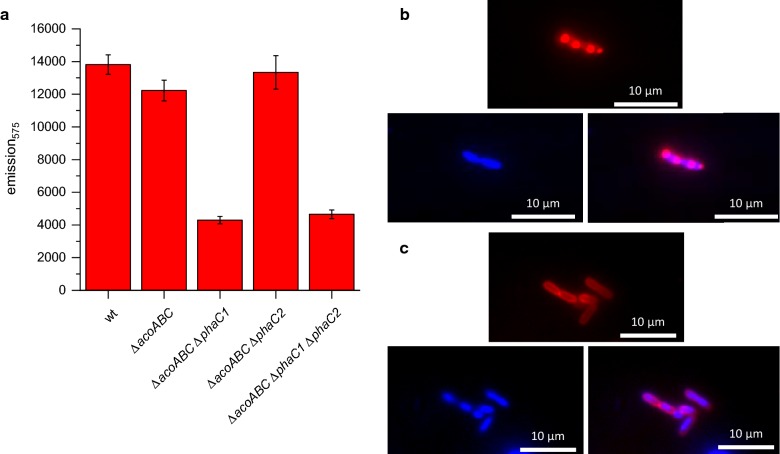
PHB production in different deletion strains. **a** Emission measurement at 575 nm after Nile Red staining and absorption of 530 nm. **b** Fluorescent microscopy pictures of *C. necator* H16 wild type after Nile Red (up) and DAPI straining (left), merged picture (right). **c** Fluorescent microscopy pictures of *C. necator* H16_Δ*acoABC_*Δ*phaC1_*Δ*phaC2*

We hypothesized that it could be possible to increase the acetoin yield of the PHB promoter system, if the two PHB synthase genes (*phaC1* and *phaC2*) were deleted from the genome as this would render the strain incapable of producing storage polymers [2]. In order to verify the influence of the different deletions on the PHB production, *C. necator* H16 wild type, *C. necator* H16_Δ*acoABC*, *C. necator* H16_Δ*acoABC*_*phaC1*, *C. necator* H16_Δ*acoABC*_*phaC2*, and *C. necator* H16_Δ*acoABC*_*phaC1*_*phaC2* were stained with Nile Red. The deletion of *phaC1* had an impact on PHB production as the Δ*phaC1* and Δ*phaC1*_Δ*phaC2* both presented an approximately 2.5-fold lower Nile Red-based emission than the strains without a deletion of *phaC1* (Fig. 3a). This result is corroborated by fluorescent microscopy experiments. Only strains containing the *phaC1* gene produced PHB granula under the chosen growth conditions. These results are in agreement with a study by Peplinski and colleagues. The authors discovered that PhaC1 was the only active PHB synthase and that even a constitutive expression of *phaC2* could not complement the mutation [12].

**Fig. 4:**
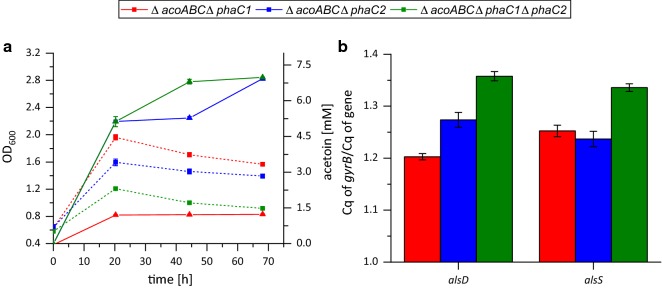
Influence of the *phaC* genes on acetoin production with the plasmid pKRphb-*alsSD* under heterotrophic growth conditions. The three different strains (*C. necator* H16_Δ*acoABC_*Δ*phaC1*, *C necator* H16_Δ*acoABC_*Δ*phaC2*, and *C. necator* H16_Δ*acoABC_*Δ*phaC1_*Δ*phaC2*) with the plasmid pKRphb-*alsSD* were characterized for growth, acetoin production, and *alsSD* expression levels. **a** Acetoin concentration (lines + up-facing triangles) and growth (dotted lines + squares). **b** Expression levels of *alsD* and *alsS* normalized to *gyrB* after 20 h

Although the *phaC1* promoter-based expression led to rather low yields in the first experiments, we repeated the heterotrophic experiments using the *phaC1* and *phaC2* deletion strains as the presence of the *phaC* genes and their gene products might affect promoter activity. Therefore, the plasmid pKRphb-*alsSD* was introduced in the strains with either one or two deletions in the PHB synthases in order to direct the carbon flux toward acetoin production. The strain with the best growth was *C. necator* H16_Δ*acoABC_*Δ*phaC1* followed by *C. necator* H16_Δ*acoABC_*Δ*phaC2* (Fig. 4a). The triple deletion mutant (*C. necator* H16_Δ*acoABC_*Δ*phaC1_*Δ*phaC2*) showed the lowest increase in OD_600_. Acetoin production showed the opposite order, and the triple deletion mutant produced the highest acetoin concentration with 6.98 mM.

Quantitative reverse transcription PCR (qRT-PCR) was conducted on the *alsSD* genes in order to find a potential correlation between expression level and acetoin yield. The quantified expression levels were normalized to the housekeeping gene *gyrB* for each test strain. The highest transcription levels for *alsS* and *alsD* were measured for the Δ*phaC1_*Δ*phaC2* double deletion mutant (Fig. 4b). *alsS* expression did not differ significantly between the Δ*phaC1* and Δ*phaC2* mutants, but *alsD* expression was lower in the Δ*phaC1* compared to Δ*phaC2* mutant. Hence, the expression values corresponded well to acetoin production. The acetoin yield from the strains with the best production with a deletion of either the Δ*phaC2* gene alone or Δ*phaC1* and Δ*phaC2* was 64.6% and 63.0% (mol/mol).

### Autotrophic acetoin production

**Fig. 5:**
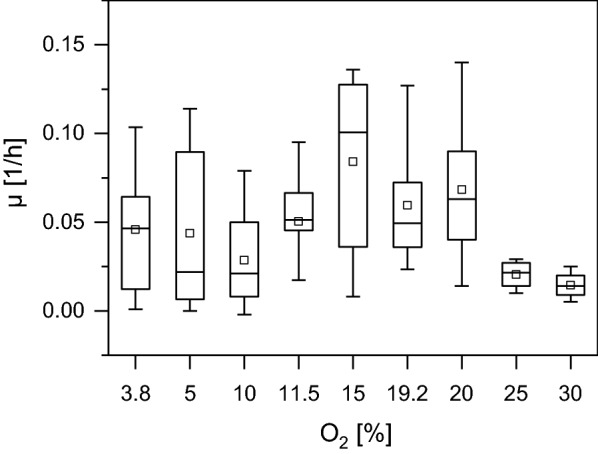
Boxplot displaying the growth coefficient for different oxygen concentrations under autotrophic conditions. The small square in the middle of the boxes indicates the average growth rate while the horizontal line indicates the growth rate’s median

Before testing the strains’ capability for autotrophic acetoin production, we aimed to elucidate the optimal oxygen concentration for lithoautotrophic growth. The experiments were conducted in 1 l fermenters with a continuous gas flow. The reactors were inoculated with *C. necator* H16 wild type to a starting OD_600_ of 0.1. The increase in O_2_ concentration from 3.8 to 20% had no significant influence on the growth rate, while concentrations > 25% seemed to inhibit growth. The boxplot indicates that the average growth rate was highest between 15 and 20% O_2_. Nevertheless, it appears that the standard deviation in most experiments was rather high (Fig. 5).

**Fig. 6:**
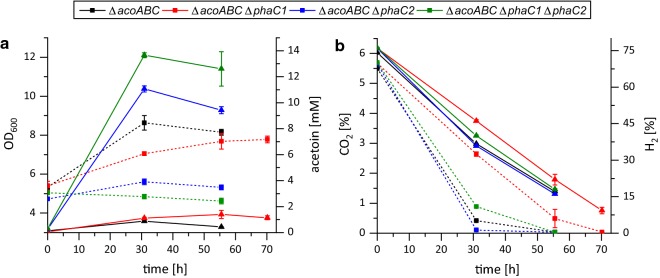
Autotrophic acetoin production in batch experiments. The four different strains (*C. necator* H16_Δ*acoABC*, *C. necator* H16_Δ*acoABC_*Δ*phaC1*, *C. necator* H16_Δ*acoABC_*Δ*phaC2*, and *C. necator* H16_Δ*acoABC_*Δ*phaC1_*Δ*phaC2*) with the plasmid pKRphb-*alsSD* were characterized for growth and acetoin production in addition to CO_2_ and H_2_ consumption. **a** Growth (dotted lines + squares) and acetoin concentration (lines + up-facing triangles). **b** CO_2_ concentration (dotted lines + squares) and H_2_ concentration (lines + up-facing triangles)

As a result of the experiments with different oxygen concentrations, we aimed for autotrophic acetoin production with a gas mixture of 75% H_2_:5% CO_2_:18% O_2_:2% N_2_. The experiments were repeated with the pKRphb-*alsSD* plasmid with the three different *phaC* deletion strains. The individual triplicate experiments were stopped when the CO_2_ concentration reached values < 0.05%. As expected, the highest acetoin levels were measured with the strain *C. necator* H16_Δ*acoABC*_Δ*phaC2* (11.06 mM) and *C. necator* H16_Δ*acoABC*_Δ*phaC1*_Δ*phaC2* (13.66 mM). Acetoin production corresponded to low or no growth, indicating low anabolic CO_2_ consumption in both strains. While H_2_ and O_2_ were consumed by all strains with similar rates, the *phaC1* deletion mutant lacked behind the others with respect to CO_2_ consumption. The acetoin yields for *C. necator* H16_Δ*acoABC* and *C. necator* H16_Δ*acoABC*_Δ*phaC1* were low with values of 6.82% and 12.03% (mol C/mol C), respectively. For the strains with the *phaC2 deletion*, the acetoin yield was determined to be 90.77% and 129.31% (*C. necator* H16_Δ*acoABC*_Δ*phaC2* and *C. necator* H16_Δ*acoABC*_Δ*phaC1*_Δ*phaC2*, respectively) (mol C/mol C) (Fig. 6).

**Fig. 7:**
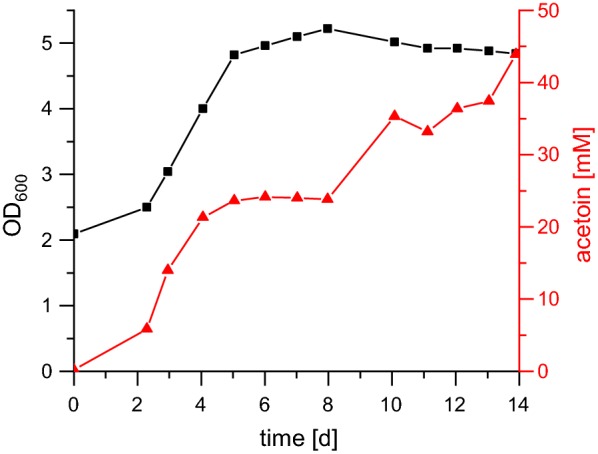
Autotrophic acetoin production under continuous growth conditions. *C. necator* H16_Δ*acoABC_*Δ*phaC1_*Δ*phaC2*_pKRphb-*alsSD* was inoculated to an OD_600_ of 2.0. The gas mixture of 80% H_2_:5% CO_2_:15% O_2_ was applied with a flow of 4 l/h for 2 weeks. Black squares: OD_600_, red triangles: acetoin

The best strain for acetoin production developed in this study was *C. necator* H16_Δ*acoABC*_Δ*phaC1*_Δ*phaC2*_pKRphb-*alsSD*, which was used in a fermenter with a continuous supply of the autotrophic gas mixture for 2 weeks. The starting OD_600_ was 2.0, and this strain grew up to an OD_600_ of around 5.0 (Fig. 7). The acetoin concentration increased to a final value of 44 mM (3.9 g/l).

## Discussion

The development of strains and processes for biotechnological production of platform chemicals from CO_2_ is a necessity for the realization of a bioeconomy. Knallgas bacteria, such as *C.* *necator*, offer the possibility to produce more complex chemicals from CO_2_ as the Calvin cycle leads to the production of C3 units, and aerobic respiratory metabolism delivers a sufficient amount of energy. Hence, this study does not only describe how efficient acetoin can be produced from CO_2_, but furthermore, it is a proof of principle for a multitude of different production processes branching from pyruvate.

Acetoin is a potential carbon source for *C.* *necator*, and the initially conducted experiments proved the necessity to delete the *acoABC* cluster if acetoin is chosen to be the final product of the process. The PHB promoter system led to rather inefficient acetoin production under heterotrophic conditions compared to the rhamnose- and arabinose-inducible promoters. This situation changed after deletion of the *phaC2* gene although the gene product did not seem to be involved in PHB production under the study’ experimental conditions. Nevertheless, PhaC2 seems to negatively influence PHB promoter activity. This is also corroborated by the conducted quantitative analysis of *alsSD* expression, which revealed that the strain with the *phaC2* deletion had higher *alsD* transcript abundance compared to the *phaC1* deletion mutant. Still, the differences in the transcription levels between the three strains were moderate, and it seems unlikely that they alone can be the reason for the observed differences. It is also unlikely that PHB production funnels too much carbon away from acetoin synthesis as the *phaC2* single deletion mutant produced similar amounts of PHB compared to the wild type. Hence, the influence of *phaC2* and/or its gene product on the activity of the PHB promoter warrants further investigation in future research studies.

It was surprising to observe that the developed inducer-independent expression system led to very high carbon efficiencies in the *phaC2* single and *phaC1_phaC2* double deletion strains. The high efficiencies corresponded well with the observed lack of growth, which suggests that CO_2_ was used to a minor extent for anabolic purposes. Carbon recovery of > 100% mol C/mol C for the double mutant was most likely the result of catabolism as the optical density in the medium decreased slightly. In the long-term production experiment, a phase of 2 days in which growth was limited was followed by rapid growth of the organism for 3 days and a rather static cell density until the end of the experiment. The transition from exponential growth to the stationary phase marked the only period within the experiment in which the acetoin concentration remained rather stable. Hence, the developed constitutive PHB promoter system seems to be suitable for a stable long-term production of chemicals in *C. necator*.

Interestingly, not only the carbon but also hydrogen efficiencies were rather high. CO_2_ reduction to acetoin necessitates the addition of 20 mols of electrons per mol of acetoin produced. We observed a hydrogen consumption that was only 2.3-fold higher than the theoretical maximum (Table 1). As previously described, there are only a limited number of studies that would allow a comparison of efficiencies. Przybylski et al. [8] gained in their study a 2-hydroxyisobutyric acid production with a carbon recovery of 0.03 mol of 2-hydroxyisobutyric per mol of CO_2_. Similar efficiencies were gained in the constructed Δ*acoABC* strain containing the pKRphb-*alsSD* plasmid. It was the further deletion of the *phaC2* gene in this strain that led to significantly increased carbon efficiency to values of 0.23 and 0.32 for the *phaC2* single deletion and the *phaC1_phaC2* double mutants, respectively.

**Table 1:** Overview of the different production rates obtained in this study

Strain *C. necator* H16	Plasmid	Induction	mol acetoin/mol fructose	mol acetoin/mol CO_2_	mol H_2_/mol acetoin
Δ*acoABC*	pKRara-*alsSD*		0.06 ± 0.01		
Δ*acoABC*	pKRara-*alsSD*	1.5 mM arabinose	0.87 ± 0.01		
Δ*acoABC*	pKRrha-*alsSD*		0.03 ± 0.00		
Δ*acoABC*	pKRrha-*alsSD*	1 mM rhamnose	0.89 ± 0.02		
Δ*acoABC*	pKRphb-*alsSD*		0.26 ± 0.01	0.02 ± 0.00	432.69 ± 0.06
Δ*acoABC_*Δ*phaC1*	pKRphb-*alsSD*		0.13 ± 0.01	0.03 ± 0.01	346.68 ± 0.20
Δ*acoABC_*Δ*phaC2*	pKRphb-*alsSD*		0.65 ± 0.00	0.23 ± 0.01	31.86 ± 0.03
Δ*acoABC_*Δ*phaC1_*Δ*phaC2*	pKRphb-*alsSD*		0.63 ± 0.00	0.32 ± 0.00	23.18 ± 0.02

Yields in mol acetoin/mol fructose (theoretical maximum 1 mol/mol) refer to the heterotrophic experiments while the yields in mol acetoin/mol CO_2_ (theoretical maximum 0.25 mol/mol) and mol H_2_/mol acetoin (theoretical minimum 10 mol/mol) were calculated from the experimental data of the autotrophic experiments

The literature provides strategies for accelerating autotrophic growth of *C. necator* by adjusting the expression levels of the organism’s different carboanhydrases to an optimum level [26]. Since this accelerated growth will most likely be due to a higher intracellular CO_2_ concentration, it seems likely that this will also lead to an increase in the RuBisCO efficiency. Hence, our next goal will be to advance hydrogen efficiency even further by adjusting the carboanhydrase expression according to the process conditions.

## Conclusion

Overall, this study revealed that *C. necator* can be used for efficient bioproduction of platform chemicals although it uses the Calvin cycle and the high energy gain of the catalyzed knallgas reaction could lead to high anabolic rates. Moreover, the presented constitutive expression system will be a step toward a very cost efficient and long-term stable production system.

## Additional files

**Additional file 1: Table S1.** Bacterial strains and plasmids used in this study. **Table S2.** Composition of the minimal media 81 (DSMZ). **Table S3.** Primers used in this study for cloning. **Table S4.** Primers used in this study for qPCR. **Table S5.** Gas mixtures and gas flows used in this study. The indicated values were used to determine the optimal oxygen concentration.

**Additional file 2.** Codon optimization of the *alsS* gene from *Bacillus subtilis* to the codon usage of *C. necator* H16. Shown is an alignment of the developed gene sequence against the original *B. subtilis * gene.

**Additional file 3.** Codon optimization of the *alsD* gene from *Bacillus subtilis* to the codon usage of *C. necator* H16. Shown is an alignment of the developed gene sequence against the original *B. subtilis * gene.

**Additional file 4: Figure S1.** Optimal concentration of arabinose as inductor for acetoin production. *C. necator* H16_Δ*acoABC*_pKRara-*alsSD* cells were induced with 0, 1, 5, 10, 50 µM, 0.1, 0.5, 1, 1.5, 3, 5, 10 mM arabinose. A. OD measurement. B. acetoin concentration. **Figure S2.** Optimal concentration of rhamnose as inductor for acetoin production. *C. necator* H16_Δ*acoABC*_pKRrha-*alsSD* cells were induced with 0, 1, 5, 10, 50 µM, 0.1, 0.5, 1, 1.5 mM rhamnose. A. OD measurement. B. acetoin concentration. **Figure S3.** Schematic illustration of the fermenter for cultivation under autotrophic conditions. The gases H_2_ (red), O_2_ (blue) and CO_2_ (gray) were mixed according to the desired end concentrations in the gas mixer (green). The gas passed a 0.2-µm filter and was pumped into the fermenter. A sensor (orange) was installed to measure pH and temperature. Samples were taken through a septum (yellow). The gas mixer and the fermenter were installed in a fume cabinet. **Figure S4.** Central carbon metabolism of *C. necator*. Fructose and CO_2_ as carbon sources and acetoin as end product are highlighted in bold. Deleted or partially deleted genes are indicated in red (^15^) or orange (^30^) arrows, respectively. Introduced genes are shown in green (^13^ and ^14^). “Phospho” and “phosphate” are abbreviated by “P”. ^1^Fructokinase, ^2^Glucose-6-P isomerase, ^3^Glucose-6-P 1-dehydrogenase, ^4^6-P-gluconolactanase, ^5^P-gluconate dehydrogenase, ^6^2-keto-3-deoxy-6-P-gluconate aldolase, ^7^Glycerinaldehyde-3-P dehydrogenase, ^8^P-glycerate kinase, ^9^P-glycerate mutase, ^10^Enolase, ^11^Pyruvate kinase, ^12^Pyruvate dehydrogenase, ^13^Acetolactate synthase, ^14^Acetolactatede carboxylase, ^15^Acetoinoxido reductase, ^16^Ribulose-5-P kinase, ^17^Ribulose-1,5-bis-P carboxylase/oxygenase, ^18^Acetaldehyde dehydrogenase, ^19^Acetyl-CoA synthetase, ^20^Citrate synthase, ^21^Aconitase, ^22^Isocitrate dehydrogenase, ^23^Ketoglutarate dehydrogenase, ^24^Succinyl-CoA synthetase, ^25^Succinic dehydrogenase, ^26^Fumarase, ^27^Malate dehydrogenase, ^28^Ketothiolase, ^29^Acetoacetyl-CoA reduktase, ^30^Polyhydroxybutyryl synthase, ^31^Polyhydroxybutyryl depolymerase and ^31^β-Oxidation.

## Data Availability

The datasets used and/or analyzed during the current study are available from the corresponding author on reasonable request.
