# Supplementary material for: Efficient biochemical production of acetoin from carbon dioxide using Cupriavidus necator H16
Source: Biotechnol Biofuels. 2019 Jun 28;12:163. doi: 10.1186/s13068-019-1512-x (PMC6598341; doi:10.1186/s13068-019-1512-x)
Supplement: Supplementary file 1 — Additional file 1: Table S1. Bacterial strains and plasmids used in this study. Table S2. Composition of the minimal media 81 (DSMZ). Table S3. Primers used in this study for cloning. Table S4. Primers used in this study for qPCR. Table S5. Gas mixtures and gas flows used in this study. The indicated values were used to determine the optimal oxygen concentration. [file 13068_2019_1512_MOESM1_ESM.docx]

**Additional tables**

**Efficient biochemical production of acetoin from carbon dioxide using *Cupriavidus necator* H16**

**Carina Windhorst ^1^ and Johannes Gescher ^1,2 *^**

^1^ Institute for Applied Biosciences, Department of Applied Biology, Karlsruhe Institute of Technology, Karlsruhe, Germany

^2^ Institute for Biological Interfaces, Karlsruhe Institute of Technology, Eggenstein-Leopoldshafen, Germany

**^*^ Correspondence:**

Johannes Gescher

[johannes.gescher@kit.edu](mailto:johannes.gescher@kit.edu)

Table S1: Bacterial strains and plasmids used in this study.

| **Strains/Plasmids** | **Relevant Genotype** | **Reference** |
| --- | --- | --- |
| *Cupriavidus necator* | | |
| H16 |  | DSM 428 |
| H16_Δ*acoABC* | Δ*acoABC* | This study |
| H16_Δ*phaC1* | Δ*phaC1* | This study |
| H16_Δ*phaC2* | Δ*phaC2* | This study |
| H16_Δ*acoABC_*Δ*phaC1* | Δ*acoABC*, Δ*phaC1* | This study |
| H16_Δ*acoABC_*Δ*phaC2* | Δ*acoABC*, Δ*phaC2* | This study |
| H16_Δ*phaC1_*Δ*phaC2* | Δ*phaC1*, Δ*phaC2* | This study |
| H16_Δ*acoABC_*Δ*phaC1_*Δ*phaC2* | Δ*acoABC*, Δ*phaC1*, Δ*phaC2* | This study |
|  | | |
| *Escherichia coli* | | |
| WM3064 | *thrB1004* *pro* *thi* *rpsL* *hsdS* *lacZ* Δ*M15RP4*–1360 Δ(*araBAD*)567 *ΔdapA1341*::[*erm* *pir*(wt)] | W. Metcalf, University of Illinois |
|  | | |
| Plasmids | | |
| pMQ150 | *km^R^*, r6k, *sacB* | [1] |
| pMQ150-Δ*acoABC* | *km^R^*, r6k, *sacB*, 500 bp upstream of *acoABC* + *Xho*I + 500 bp downstream of *acoABC* | This study |
| pMQ150-Δ*phaC1* | *km^R^*, r6k, *sacB*, 500 bp upstream of *phaC1* + *Xho*I + 500 bp downstream of *phaC1* | This study |
| pMQ150-Δ*phaC2* | *km^R^*, r6k, *sacB*, 500 bp upstream of *phaC2* + *Eco*RI + 500 bp downstream of *phaC2* | This study |
| pKRrha-eGFP | *tc^R^*, P_rha_, RSF1010 *mob* and origin of replication, *par*, *rhaR*, *rhaS*, eGFP | [2] |
| pKRrha-*alsSD* | *tc^R^*, P_rha_, RSF1010 *mob* and origin of replication, *par*, *rhaR*, *rhaS*, *alsSD* (codon optimized*) | This study |
| pKRara-*alsSD* | *tc^R^*, P_ara_, RSF1010 *mob* and origin of replication, *par*, araC, *alsSD* (codon optimized*) | This study |
| pKRphb-*alsSD* | *tc^R^*, P_phb_, RSF1010 *mob* and origin of replication, *par*, *alsSD* (codon optimized*) | This study |
| pBAD202 | *km^R^*, P_ara_ | Invitrogen (Karlsruhe, Germany) |
| pMAT-*alsSD* I | *amp^R^*, *alsSD* part 1 (codon optimized*) | Invitrogen (Karlsruhe, Germany) |
| pT7-*alsSD* II | *amp^R^*, *alsSD* part 2 (codon optimized*) | This study |

* Sequence is available in the supplements (supplements Sequence *alsS* und Sequence *alsD*).

1. Shanks RMQ, Kadouri DE, MacEachran DP, O’Toole GA. New yeast recombineering tools for bacteria. Plasmid. 2009;62:88–97.

2. Sydow A, Pannek A, Krieg T, Huth I, Guillouet SE, Holtmann D. Expanding the genetic tool box for *Cupriavidus necator* by a stabilized L-rhamnose inducible plasmid system. J Biotechnol. Elsevier; 2017;263:1–10.

Table S2: Composition of the minimal media 81 (DSMZ).

| **Component** | **Amount per 1 l** |
| --- | --- |
| NH_4_Cl | 1 g |
| MgSO_4_ * 7 H_2_O | 0.5 g |
| CaCl_2_ * 2 H_2_O | 10 mg |
| MnCl_2_ * 4 H_2_O | 65 mg |
| NaVO_3_ * H_2_O | 5 mg |
| ZnSO_3_ * 7 H_2_O | 5 mg |
| H_3_BO_4_ | 15 mg |
| CoCl_2_ * 6 H_2_O | 10 mg |
| CuCl_2_ * 2 H_2_O | 0.5 mg |
| NiCl_2_ * 6 H_2_O | 1 mg |
| Na_2_MoO_4_ * 2 H_2_O | 1.5 mg |
| KH_2_PO_4_ | 2.3 g |
| Na_2_HPO_4_ * 2 H_2_O | 2.9 g |
| FeNH_4_ Citrate | 50 mg |
| NaHCO_3_ | 0.5 g |
| Riboflavin | 0.5 mg |
| Thiamine-HCl * 2 H_2_O | 2.5 mg |
| Nicotinic acid | 2.5 mg |
| Pyridoxine-HCl | 2.5 mg |
| Ca-Pantothenate | 2.5 mg |
| Biotin | 5 µg |
| Folic acid | 10 µg |
| Vitamin B12 | 50 µg |

Table S3: Primers used in this study for cloning.

| **Primer** | **Sequence (5’ -> 3’)** |
| --- | --- |
| 188 | GGCGGCAATGGATGCGTAC |
| 2581 | CAAAGACCTTGACGGTTGCTCTCGAGGTCTGTCTCCTTCGTTTTCAGG |
| 2588 | TGGTCGATGTCTGCGATG |
| 2589 | GGCGACCGCACCATC |
| 2596 | TAAACAAAAAGAGTTTGTAGAAACGCAAAAAGGCCATCCGTCAGGATGGCCTGTGGAAGACCGACACG |
| 2597 | TGGTTCCGCGCACATTTCCCCGAAAAGTGCCACCTAAATTGTAAGCGTTATGAGCGCATCGAAGTACG |
| 2636 | CCTGAAAACGAAGGAGACAGACCTCGAGAGCAACCGTCAAGGTCTTTG |
| 2657 | ATGAACAATTCTTAAGAAGGAGATATACATATGCTGACCAAGGCCACC |
| 2658 | TCTTCTCTCATCCGCCAAAACAGCCAAGCTTTATTCAGGCGAGCCTTCG |
| 2682 | AGCTTGCATGCCTGCAGGTCGACTCTAGAGGTGATCGCCATCATCAGC |
| 2683 | ACACAGGAAACAGCTATGACCATGATTACGAGCTTGGCATCGCCC |
| 2684 | CGGCAGAGAGACAATCAAATCCTCGAGCGCTTGCATGAGTGCC |
| 2685 | GGCACTCATGCAAGCGCTCGAGGATTTGATTGTCTCTCTGCCG |
| 2687 | AGCTTGCATGCCTGCAGGTCGACTCTAGAGGTCGACGTGACGGACTTCG |
| 2688 | CGGGCAACACCTGCAGAATTCCCTCTTGTTCACTGTGCTGCG |
| 2689 | CGCAGCACAGTGAACAAGAGGGAATTCTGCAGGTGTTGCCCG |
| 2690 | ACACAGGAAACAGCTATGACCATGATTACGTCTGAAGGGGACATGGGTTTC |
| 2693 | GCATAAAAACTGTTGTAATTCATTAAGCATTC |
| 2694 | TGGTCGCGTTCAAGGTACG |
| 2695 | CTTCTGTTCCTTGGTGGCCTTGGTCAGCATGATTTGATTGTCTCTCTGCCGTCAC |
| 2696 | ATGCTTAATGAATTACAACAGTTTTTATGCGTGTGGGGCCGCACC |
| 2697 | ATGCTTAATGAATTACAACAGTTTTTATGCTTATGACAACTTGACGGCTACATC |
| 2698 | CTTCTGTTCCTTGGTGGCC |
| 2701 | ATGCTGACCAAGGCCACCAAG |

Table S4: Primers used in this study for qPCR.

| **Gene** | **Sequence for** | **Sequence rev** | **Length [bp]** |
| --- | --- | --- | --- |
| *alsS* | AAGAAGTACAACCGCACC | GACCTTCTGCGTTCATGC | 145 |
| *alsD* | GCGAATCGAACATCCAGG | GGGTGAAGAAGGTGAACG | 285 |
| *gyrB* | ACGACGCTAGATGTGACCC | TCAAACATCAATATTCCGCGCC | 159 |
| negative | CGCCGTTGTGTTCCTTACC | AACTTCCGTCGTCTTCGC | 178 |

Table S5: Gas mixtures and gas flows used in this study. The indicated values were used to determine the optimal oxygen concentration.

| **4 l/h** | | | **5.2 l/h** | | |
| --- | --- | --- | --- | --- | --- |
| **H_2_ [%]** | **CO_2_ [%]** | **O_2_ [%]** | **H_2_ [%]** | **CO_2_ [%]** | **O_2_ [%]** |
| 90 | 5 | 5 | 76.9 | 19.2 | 3.8 |
| 85 | 5 | 10 | 76.9 | 11.5 | 11.5 |
| 80 | 5 | 15 | 76.9 | 3.9 | 19.2 |
| 75 | 5 | 20 | 85 | 5 | 10 |
| 70 | 5 | 25 | 75 | 5 | 20 |
|  |  |  | 65 | 5 | 30 |
