# Supplementary material for: Efficient biochemical production of acetoin from carbon dioxide using Cupriavidus necator H16
Source: Biotechnol Biofuels. 2019 Jun 28;12:163. doi: 10.1186/s13068-019-1512-x (PMC6598341; doi:10.1186/s13068-019-1512-x)
Supplement: Supplementary file 4 — Additional file 4: Figure S1. Optimal concentration of arabinose as inductor for acetoin production. C. necator H16_ΔacoABC_pKRara-alsSD cells were induced with 0, 1, 5, 10, 50 µM, 0.1, 0.5, 1, 1.5, 3, 5, 10 mM arabinose. A. OD measurement. B. acetoin concentration. Figure S2. Optimal concentration of rhamnose as inductor for acetoin production. C. necator H16_ΔacoABC_pKRrha-alsSD cells were induced with 0, 1, 5, 10, 50 µM, 0.1, 0.5, 1, 1.5 mM rhamnose. A. OD measurement. B. acetoin concentration. Figure S3. Schematic illustration of the fermenter for cultivation under autotrophic conditions. The gases H2 (red), O2 (blue) and CO2 (gray) were mixed according to the desired end concentrations in the gas mixer (green). The gas passed a 0.2-µm filter and was pumped into the fermenter. A sensor (orange) was installed to measure pH and temperature. Samples were taken through a septum (yellow). The gas mixer and the fermenter were installed in a fume cabinet. Figure S4. Central carbon metabolism of C. necator. Fructose and CO2 as carbon sources and acetoin as end product are highlighted in bold. Deleted or partially deleted genes are indicated in red (15) or orange (30) arrows, respectively. Introduced genes are shown in green (13 and 14). “Phospho” and “phosphate” are abbreviated by “P”. 1Fructokinase, 2Glucose-6-P isomerase, 3Glucose-6-P 1-dehydrogenase, 46-P-gluconolactanase, 5P-gluconate dehydrogenase, 62-keto-3-deoxy-6-P-gluconate aldolase, 7Glycerinaldehyde-3-P dehydrogenase, 8P-glycerate kinase, 9P-glycerate mutase, 10Enolase, 11Pyruvate kinase, 12Pyruvate dehydrogenase, 13Acetolactate synthase, 14Acetolactatede carboxylase, 15Acetoinoxido reductase, 16Ribulose-5-P kinase, 17Ribulose-1,5-bis-P carboxylase/oxygenase, 18Acetaldehyde dehydrogenase, 19Acetyl-CoA synthetase, 20Citrate synthase, 21Aconitase, 22Isocitrate dehydrogenase, 23Ketoglutarate dehydrogenase, 24Succinyl-CoA synthetase, 25Succinic dehydrogenase, 26Fumarase, 27Malate dehydrogenase, 28Ketothiolase, 29 [file 13068_2019_1512_MOESM4_ESM.docx]

**Additional figures**

**Efficient biochemical production of acetoin from carbon dioxide using *Cupriavidus necator* H16**

**Carina Windhorst ^1^ and Johannes Gescher ^1,2 *^**

^1^ Institute for Applied Biosciences, Department of Applied Biology, Karlsruhe Institute of Technology, Karlsruhe, Germany

^2^ Institute for Biological Interfaces, Karlsruhe Institute of Technology, Eggenstein-Leopoldshafen, Germany

**^*^ Correspondence:**

Johannes Gescher

[johannes.gescher@kit.edu](mailto:johannes.gescher@kit.edu)

Figure S1: Optimal concentration of arabinose as inductor for acetoin production. *C. necator* H16_Δ*acoABC*_pKRara-*alsSD* cells were induced with 0, 1, 5, 10, 50 µM, 0.1, 0.5, 1, 1.5, 3, 5, 10 mM arabinose. A. OD measurement. B. acetoin concentration.

Figure S2: Optimal concentration of rhamnose as inductor for acetoin production. *C. necator* H16_Δ*acoABC*_pKRrha-*alsSD* cells were induced with 0, 1, 5, 10, 50 µM, 0.1, 0.5, 1, 1.5 mM rhamnose. A. OD measurement. B. acetoin concentration.


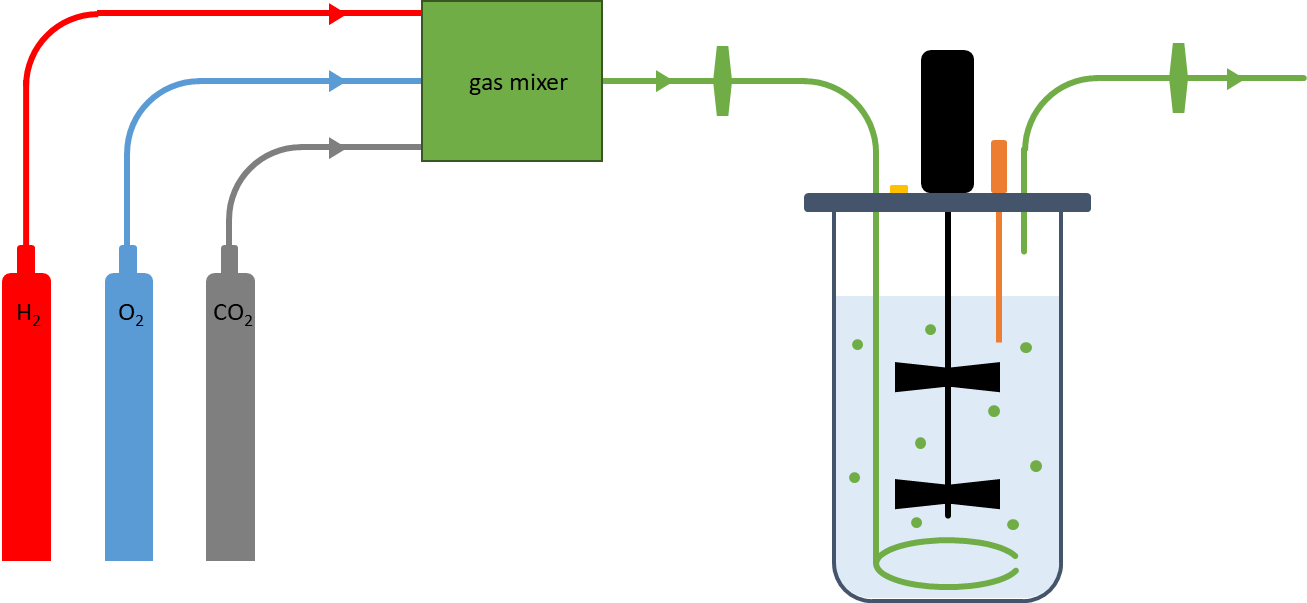


Figure S3: Schematic illustration of the fermenter for cultivation under autotrophic conditions. The gases H_2_ (red), O_2_ (blue) and CO_2_ (grey) were mixed according to the desired end concentrations in the gas mixer (green). The gas passed a 0.2 um filter and was pumped into the fermenter. A sensor (orange) was installed to measure pH and temperature. Samples were taken through a septum (yellow). The gas mixer and the fermenter were installed in a fume cabinet.

Ketoglutarate

Acetaldehyde

**Fructose**

Fructose-6-P

Glucose-6-P

Glucono-1,5-lacton-6-P

6-P-gluconate

2-keto-3-deoxy-6-P-gluconate

^1^

^2^

^3^

^4^

^5^

^6^

Pyruvate

1,3-bis-P-glycerate

3-P-glycerate

2-P-glycerate

P-enolpyruvate

Glycerinaldehyde-3-P

^7^

^8^

^9^

^10^

Acetolactate

**Acetoin**

Acetyl-CoA

^11^

^12^

^13^

^14^

^15^

Ribulose-5-P

Ribulose-1,5-bis-P

^16^

^17^

Acetate

^18^

^19^

Citrate

Aconitate

Isocitrate

Succinyl-CoA

Succinate

Fumarate

Malate

Oxalacetate

^20^

^21^

^21^

^22^

^23^

^24^

^25^

^26^

^27^

3-Acetoacetyl-CoA

3-Hydroxybutyryl-CoA

Polyhydroxybutyrate

Crotonyl-CoA

^28^

^29^

^30^

^31^

^32^

**CO_2_**

Figure S4: Central carbon metabolism of *C. necator*. Fructose and CO_2_ as carbon sources and acetoin as end product are highlighted in bold. Deleted or partially deleted genes are indicated in red (^15^) or orange (^30^) arrows, respectively. Introduced genes are shown in green (^13^ and ^14^). “Phospho” and “phosphate” are abbreviated by “P”. ^1^Fructokinase, ^2^Glucose-6-P isomerase, ^3^Glucose-6-P 1-dehydrogenase, ^4^6-P-gluconolactanase, ^5^P-gluconate dehydrogenase, ^6^2-keto-3-deoxy-6-P-gluconate aldolase, ^7^Glycerinaldehyde-3-P dehydrogenase, ^8^P-glycerate kinase, ^9^P-glycerate mutase, ^10^Enolase, ^11^Pyruvate kinase, ^12^Pyruvate dehydrogenase, ^13^Acetolactate synthase, ^14^Acetolactatede carboxylase, ^15^Acetoinoxido reductase, ^16^Ribulose-5-P kinase, ^17^Ribulose-1,5-bis-P carboxylase/oxygenase, ^18^Acetaldehyde dehydrogenase, ^19^Acetyl-CoA synthetase, ^20^Citrate synthase, ^21^Aconitase, ^22^Isocitrate dehydrogenase, ^23^Ketoglutarate dehydrogenase, ^24^Succinyl-CoA synthetase, ^25^Succinic dehydrogenase, ^26^Fumarase, ^27^Malate dehydrogenase, ^28^Ketothiolase, ^29^Acetoacetyl-CoA reduktase, ^30^Polyhydroxybutyryl synthase, ^31^Polyhydroxybutyryl depolymerase and ^31^β-Oxidation.
