# Supplementary material for: Efficient biochemical production of acetoin from carbon dioxide using Cupriavidus necator H16
Source: Biotechnol Biofuels. 2019 Jun 28;12:163. doi: 10.1186/s13068-019-1512-x (PMC6598341; doi:10.1186/s13068-019-1512-x)
Supplement: Supplementary file 3 — Additional file 3. Codon optimization of the alsD gene from Bacillus subtilis to the codon usage of C. necator H16. Shown is an alignment of the developed gene sequence against the original B. subtilis gene. [file 13068_2019_1512_MOESM3_ESM.pdf]

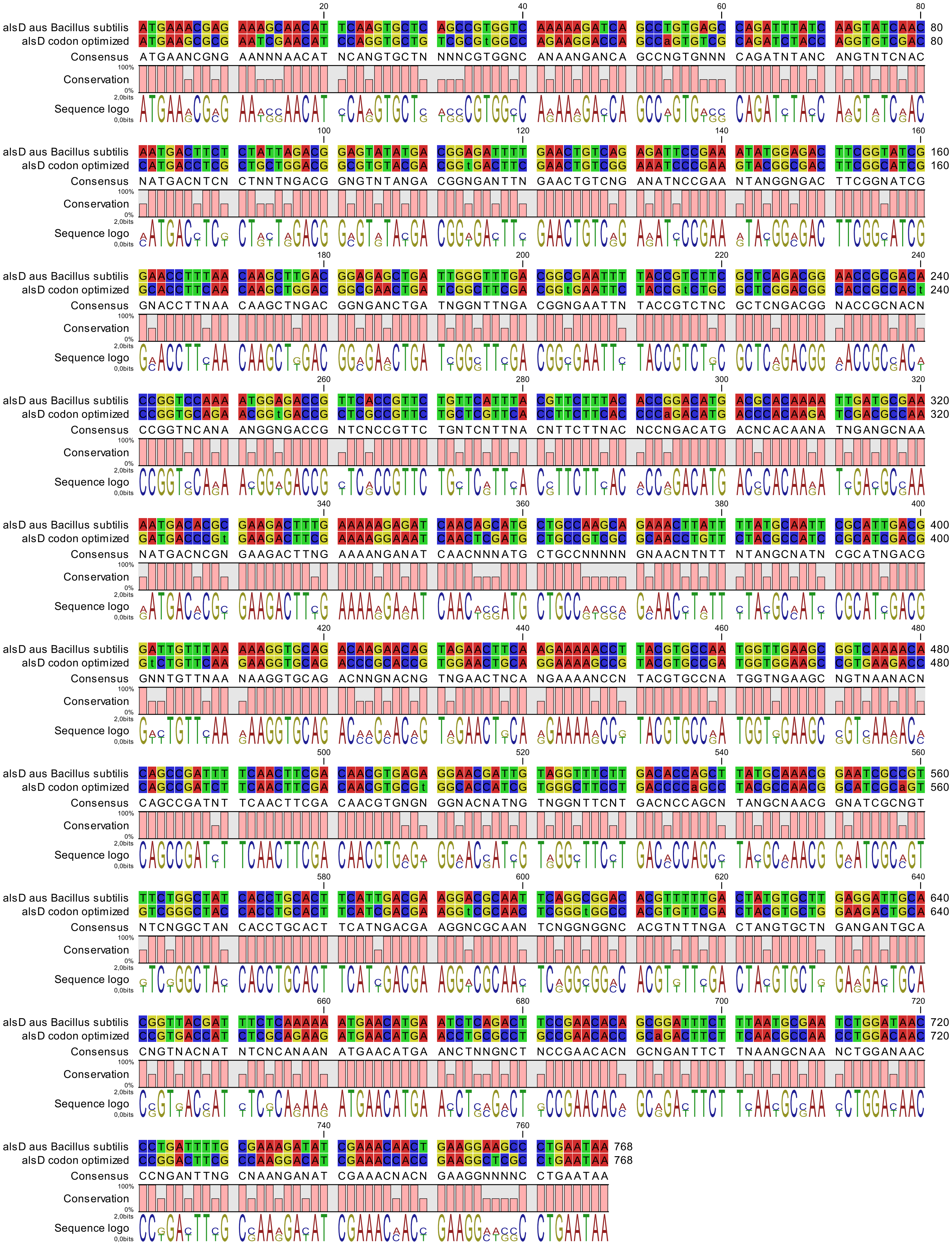
alsD aus Bacillus subtilis
alsD codon optimized
Consensus
Conservation
Sequence logo
